# Long-term clinical outcomes of everolimus-eluting stent versus paclitaxel-eluting stent in patients undergoing percutaneous coronary interventions: a meta-analysis

**DOI:** 10.1186/s12872-016-0206-6

**Published:** 2016-02-09

**Authors:** Min Meng, Bei Gao, Xia Wang, Zheng-gang Bai, Ri-na Sa, Bin Ge

**Affiliations:** Department of Pharmacy, Gansu Provincial Hospital, Donggang West Road No. 204, Lanzhou, Gansu 730000 China; Evidence-Based Medicine Center, Lanzhou University, Lanzhou, Gansu 730000 China

**Keywords:** Everolimus-eluting stent, Paclitaxel-eluting stent, Percutaneous coronary interventions, Systematic review, Meta-analysis

## Abstract

**Background:**

Everolimus -eluting stent (EES) is common used in patients undergoing percutaneous coronary interventions (PCI). Our purpose is to evaluate long-term clinical outcomes of everolimus -eluting stent (EES) versus paclitaxel-eluting stent (PES) in patients undergoing percutaneous coronaryinterventions (PCI) in randomized controlled trials (RCTs).

**Methods:**

We searched Medline, EMBASE, Cochrane Library, CNKI, VIP and relevant websites (https://scholar-google-com.ezproxy.lib.usf.edu/) for articles to compare outcomes between everolimus-eluting stent and paclitaxel-eluting stent without language or date restriction. RCTs that compared the use of everolimus -eluting stent and paclitaxel-eluting stent in PCI were included. Variables relating to patient, study characteristics, and clinical endpoints were extracted. Meta-analysis was performed using RevMan 5.2 software.

**Results:**

We identified 6 published studies (from three randomized trials) more on everolimus-eluting stent (*n* = 3352) than paclitaxel-eluting (*n* = 1639), with follow-up duration ranging from 3, 4 and 5 years. Three-year outcomes of everolimus-eluting stent compared to paclitaxel-eluting were as following: the everolimus-eluting stent significantly reduced all-cause death (relative risk [RR]:0.63; 95 % confidence interval [CI]: 0.46. to 0.82), MACE (RR: 0.56; 95 % CI: 0.41 to 0.77), MI (RR: 0.64; 95 % CI: 0.48 to 0.86), TLR (RR: 0.72; 95 % CI: 0.59 to 0.88), ID-TLR (RR: 0.74; 95 % CI: 0.59 to 0.92) and ST (RR: 0.54; 95 % CI: 0.32 to 0.90). There was no difference in TVR between the everolimus-eluting and paclitaxel-eluting (RR: 0.76; 95 % CI: 0.58 to 1.10); Four-year outcomes of everolimus-eluting compared to paclitaxel-eluting: the everolimus-eluting significantly reduced MACE (RR: 0.44; 95 % CI: 0.18 to 0.98) and ID-TLR (RR: 0.47; 95 % CI: 0.23 to 0.97). There was no difference in MI (RR: 0.48; 95 % CI: 0.16 to 1.46), TLR (RR: 0.46; 95 % CI: 0.20 to1.04) and ST ((RR: 0.34; 95 % CI: 0.05 to 2.39). Five-year outcomes of everolimus-eluting stent compared to paclitaxel-eluting: There was no difference in ID-TLR (RR: 0.67; 95 % CI: 0.45 to 1.02) and ST (RR: 0.71; 95 % CI: 0.28 to1.80).

**Conclusions:**

In the present meta-analysis, everolimus-eluting appeared to be safe and clinically effective in patients undergoing PCI in comparison to PES in 3-year clinical outcomes; there was similar no difference in reduction of ST between EES and PES in long-term(≥4 years) clinical follow-ups. Everolimus-eluting is more safety than paclitaxel-eluting in long-term clinical follow-ups, whether these effects can be applied to different patient subgroups warrants further investigation.

## Background

Acute coronary syndrome (ACS) is a major public health concern worldwide. Coronary artery disease is a leading cause of morbidity and mortality in adults around the world, and accounts for an even higher proportion of deaths in developed countries. The WHO estimates that 7.3 million people died of coronary heart disease in 2008. Moreover, the number of people who die from cardiovascular diseases, mainly from heart disease and stroke, will increase and accounts for 23.3 million by the year of 2030 [1, 2]. Furthermore, ACS bears a heavy economic burden for government and society in developed countries [3, 4], which has also drawn attention of the experts in developing countries due to the increased risk factors of cardiovascular disease, including the prevalence of hypercholesterolemia [5] and more smokers [6] etc.

Currently, percutaneous coronary intervention (PCI) is another important means of reperfusion therapy in treatment of patients with ACS, especially for patients with ST-segment elevation myocardial infarction (STEMI), with the additional drug therapies such as antiplatelet drugs, anticoagulants, stains and thrombolytic therapy etc. [7]. Compared with thrombolytic therapy, PCI is more effective in restoring coronary blood flow. Numerous studies, including a large meta-analysis showed that PCI have shown the superiority in reducing mortality, recurrent myocardial infarction and stroke compared with thrombolytic therapy while the lower risk of bleeding caused by PCI. Therefore, reperfusion therapy is regarded as the standards and is recommended for patients with STEMI [8].

Stents become more and more popular used to PCI. Since the advent of the first use of stents—Palmaz stents in the last century [9], bare metal stents (BMS) were widely used in balloon angioplasty which was the most popular method of treating heart disease and was recommended by the American Medical Association as the standard treatment [10]. But a long-term study for patients who received a single Palmaz stents showed that the incidence of restenosis was 30.2 %, while the incidence ranged from 28 % to 41 % in patients who received angioplasty alone [11]. Arterial wall damage response mechanism triggered by Balloon angioplasty and stenting, resulting in intimal hyperplasia was an important cause of restenosis [10]. In order to reduce tissue proliferation, first-generation drug-eluting stents (DES) were being designed and coated with a polymer allowing controlled local delivery of a pharmaceutical agent with antineoplastic and anti-inflammatory properties [12]. Now, recommendations of the European Society of Cardiology call for the use of drug-eluting stents for PCI if the patient has no contraindication to extended treatment with dual-antiplatelet therapy [13].

Paclitaxel-eluting stent was one of the first-generation drug eluting stents. Comparing with BMS, Paclitaxel-Eluting Stent reduced the rates of restenosis significantly. However, some studies have shown that some type of stent thrombosis occurs [14], the two large researches in first-generation DES showed that an annual rate of late stent thrombosis was 0.4–0.6 % for up to 4 years after stent implantation [15–17]. Thus, the second-generation DES was designed to reduce the incidence of late stent thrombosis and solve the problem of restenosis by replacing the coating drug.

Everolimus-eluting stent (EES) was a second generation DES approved by the FDA in July 2008 with its cobalt chromium stent design, high deliverability, and everolimus drug coating used to prevent abnormal tissue growth [18]. Due to its lipophilic chemical structure, it is more rapidly absorbed into the arterial wall, potentially making it a better drug for local intravascular delivery following stent implantation [19, 20]. The FUTURE Trail, EXAMINATION Trail [21] and SPIRIT FIRST Trail showed the EES to be safe, feasible, and efficient [14, 22–26]. The studies showed the superiority of PES to EES for short time [27–30]. Despite this, the long-term efficacy of EES use should be investigated to reduce the individual trails and the limitations of short-term studies.

Similarly, the TAXUS trials assessed the safety and efficacy of paclitaxel-eluting stent (PES) in the treatment of coronary artery disease [31–34]. Large randomized clinical trials such as SPIRIT Trail and COMPARE Trails designed to compare EES with PES have shown reduced rates of repeat revascularization, major adverse cardiac events, myocardial infarction and stent thrombosis [35–38]. Hence, it is necessary to carry out a new meta-analysis including RCTs only and to update the prior meta-analyses on the basis of the preferred reporting items for systematic reviews and meta-analysis (PRISMA) items [39].

A comprehensive network meta-analysis had displayed that cobalt-chromium everolimus eluting stents (CoCr-EES) has the lowest rate of baremetal stents, paclitaxel-eluting stents(PES), sirolimus-eluting stents, phosphorylcholine-based zotarolimuseluting stentsstent thrombosis, and Resolute zotarolimus-eluting stents within 2 years of implantation. However, the study only inculed 6 stduies with one and 2 years follow ups. The aim of this meta-analysis was to compare the efficacy and safety of EES versus PES especially with regards to the patient of all-cause death, major adverse cardiac events, stent thrombus, and myocardial infarction as primary outcomes, and of target lesion revascularization, ischemia-driven target lesion revascularization, and target vessel revascularization as secondary outcomes over long-term (followed up 3, 4 and 5 years), and to provide much more reliable evidences for clinical decision-making and to guide future research [40].

## Methods

### Protocol and registration

No protocol has been registered in public, however draft related to the study already exists.

### Search strategy and selection criteria

We searched Medline, EMBASE, Cochrane Library, CNKI, VIP, and relevant websites (https://scholar-google-com.ezproxy.lib.usf.edu/) by two researchers (Min.M. and Bei.G.). Disagreements were resolved by consensus. The reference list of relevant studies was further scanned. No restrictions of language, publication date or publication status were imposed. The last search was run on October 2014.

**Fig. 1:**
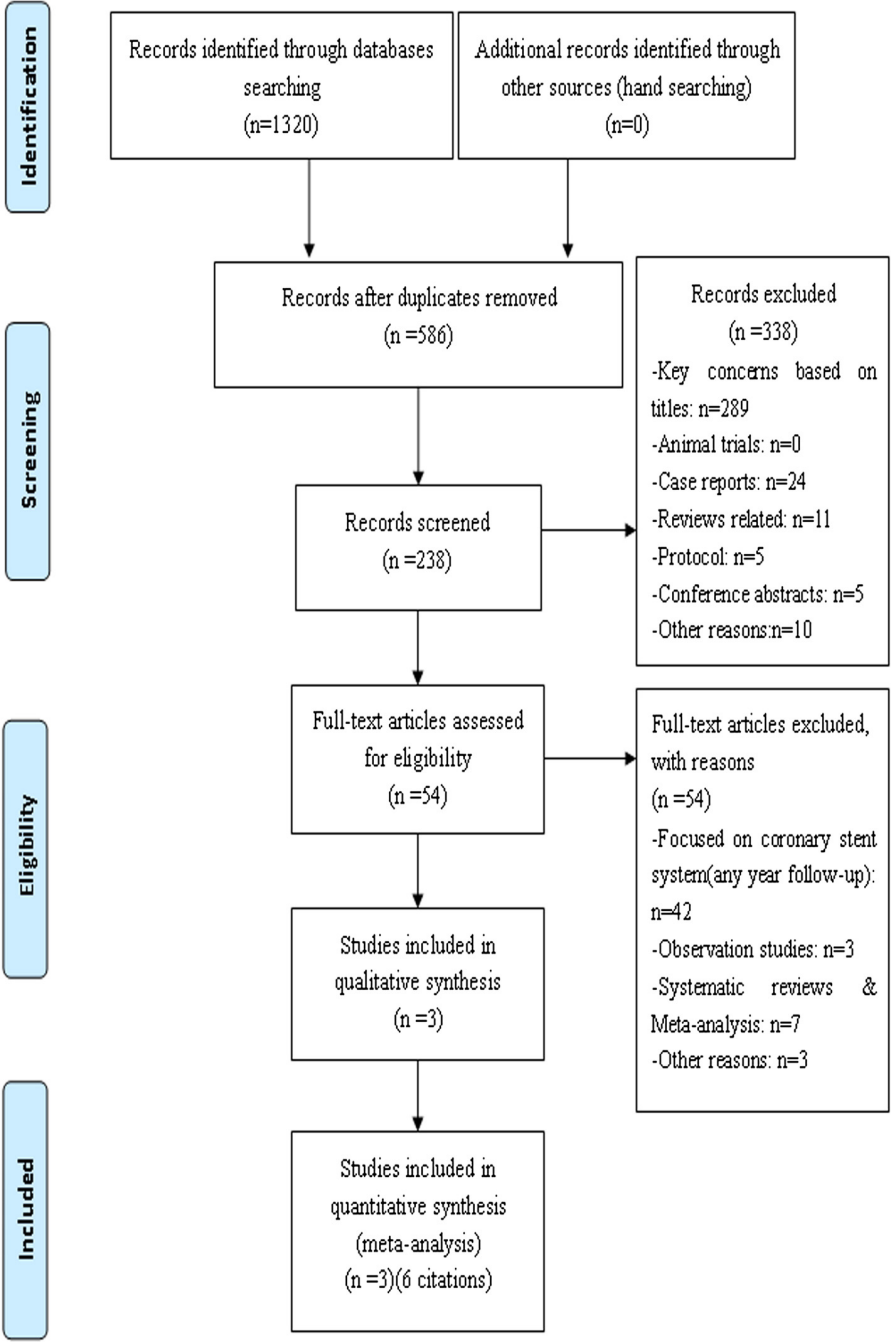
Data source flow chart diagram

The following search terms were used: “percutaneous coronary intervention”, “randomized trial”, “everolimus-eluting stent”, “Xience”, “drug-eluting stent”, “paclitaxel-eluting stent” and “TAXUS”. To be included in this study, the citation had to meet the following criteria: randomized controlled trials that compared the use of EES and PES in percutaneous coronary intervention (PCI). Exclusion criteria were: (1) ongoing studies; (2) irretrievable data; (3) Follow up duration of less than 3 years; (4) non randomized studies; (5) animal studies; (6) case reports; (7) related reviews; (8) protocols; (9) conference abstracts. The Flow Diagram was shown in Fig. 1.

### Data extraction and risk of bias assessment

Two investigators (MENG.M. and GAO.B.) independently included reports at title and/or at abstract level, disagreement was resolved with a third reviewer (GE.B.), and studies that met inclusion criteria were selected for further analysis. Furthermore, we e-mailed authors of trials for supplemental data which were partially published.

Data extraction included:

1. General information: title, authors, publication date, and article sources;
2. Study characteristics: subject characteristics, purpose, sites, study period, comparability of baseline, research results;
3. Primary Outcomes: major adverse cardiac events (MACE), myocardial infarction (MI), cardiac death, stent thrombus (ST); Secondary outcomes: target lesion revascularization (TLR), target vessel revascularization (TVR), all-cause death,, ischemia-driven target lesion revascularization (ID-TLR).

**Fig. 2:**
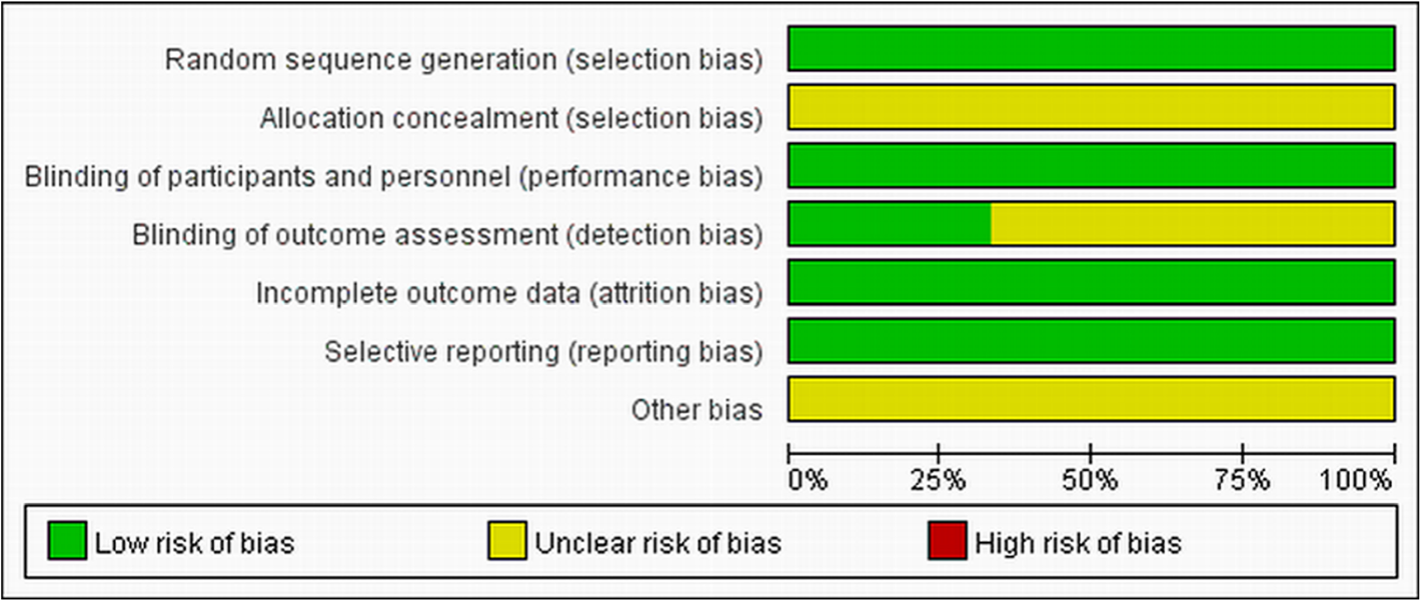
Risk of bias graph

Quality of included studies was appraised by two investigators (M.M. and G.B.), and the assessment is shown in Fig. 2. The risk of selection, performance, detection, attrition, and reporting bias (expressed as low risk of bias, high risk or unclear risk of bias, the underlying risk of bias can’t be determined due to incomplete reporting) were evaluated separately [41].

### Data analysis

We used Review Manager (5.2) software for Meta analysis, and calculated the relative risk (RR) from the abstracted data. The average effects for the outcomes and 95 % confidence intervals (CI) were obtained using a random effects mode. Heterogeneity of RR across trials was assessed using the Cochrane Q statistic (*P* value ≤ 0.05 was considered significant) and the I2 statistic.

**Fig. 3:**
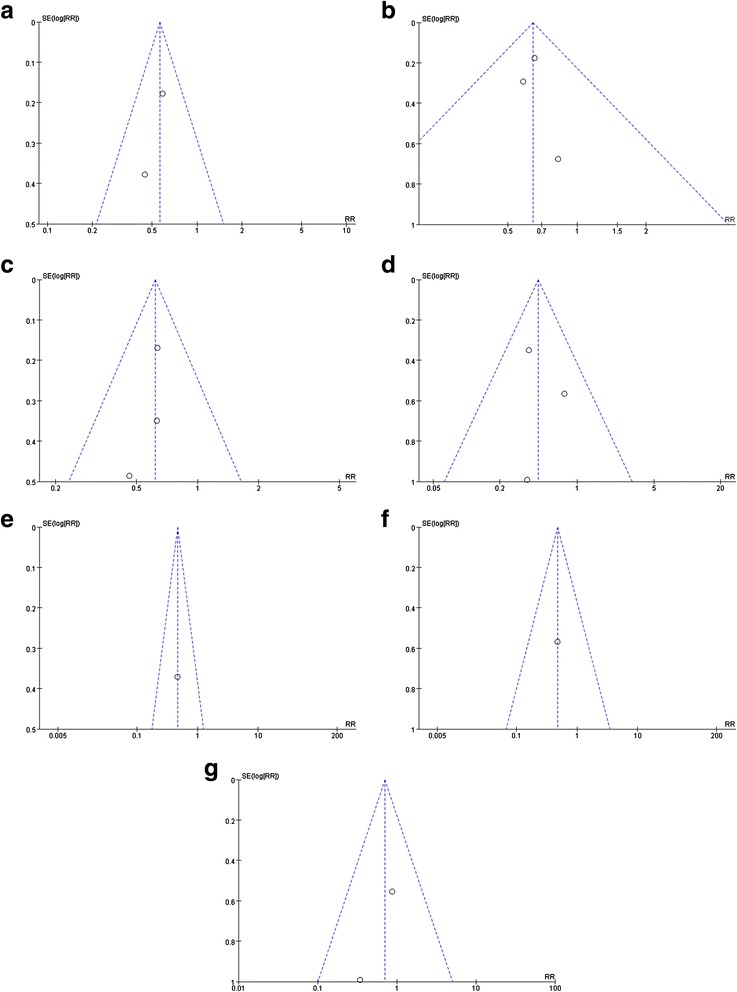
Funnel plot of primary outcomes included in the meta-analysis. The funnel plot of (**a**: 3-year MACE, **b**: 3-year MI, **c**: 3-year death, **d**: 3-year ST, **e**: 4-year MACE, **f**: 4-year MI, **g**: 5-year ST). the standard error (SE) of the ln relative risk (RR) was plotted against the relative risk for (**a**:3-year MACE, **b**: 3-year MI, **c**: 3-year death, **d**: 3-year ST, **e**: 4-year MACE, **f**: 4-year MI, **g**: 5-year ST) 3-year MACE. The absence of any asymmetric distribution suggested no publication bias

For the primary endpoint, small-study effects were analyzed by constructing a funnel plot, in which the standard error of the lnRR was plotted against RR for 3–5 years follow ups as minimize the publication bias. The absence of any asymmetric distribution suggested no publication bias. The funnel plots of primary outcomes were shown in Fig. 3(a-g).

## Results

### Search results

One thousand three hundred twenty articles were accessed through searching, and 734 articles were retrieved after duplicate were removed by Endnote X4 software, then abstracts and full texts were reviewed again with excluding duplicate papers and articles that do not meet the inclusion criteria. Finally, six published studies [42–47] from 3 randomized controlled trials were included in the present meta-analysis.

### Characteristics of the included reviews

There were more patients randomized to EES (*n* = 3352) than to PES (*n* = 1639), resulted from imbalanced randomization in certain studies. The mean age ranged from 62 to 65 years with the majority of patients being male. Diabetes were not excluded. The frequency of diabetes mellitus ranged from 23.67 % to 32.14 %. Follow-up period ranged from 3.4 to 5 years. General characteristics of the included studies are shown in Table 1.

**Table 1:** Baseline of characteristics of the included trials

Source		Number of patents	Age	Male	Diabetes	Diameter stenosis (%)	Vessel diameter (mm)	Lesion length (mm)	Inclusion criteria	Exclusion criteria	Primary outcomes	Secondary outcomes	Other outcomes	Follow-up duration, year	Funding
SPIRIT II [45–47]	EES^*^	223	62 ± 10	158	51	61 ± 12	2.70 ± 0.52	13.0 ± 5.7	1. Ischemia and vessel size 2.5–4.25 mm and lesion length ≤ 28 mm; 2. A percentage diameter stenosis (DS) 50 %−99 %	1. Recent MI^‡^,LVEF^x^ ≤ 30 %; 2. LM^§^ heavily calcified lesion; 3. Visible thrombus	.MI^‡^ ST^††^	ID-TLR^#^ ID-MACE^ll^	CABG^**^	3.4,5	Abbott vascular
	PES^†^	77	62 ± 9	61	20	59 ± 10	2.82 ± 0.58	13.2 ± 6.4							
SPIRIT III [48, 49]	EES^*^	669	63.2 ± 10.5	469	198	70.0 ± 13.3	2.77 ± 0.45	14.7 ± 5.6	1. Stable, unstable angina; 2. Ischemia with vessel size 2.5–3.75 mm and lesion length ≤ 28 mm	1. Recent MI^‡^,LVEF^x^ < 30 %; 2. LM^§^ bifurcation; 3. BG; 4. Calcification; 5. Thrombus	MAGE^‡‡^ Cardiac death MI^‡^ ST^††^	TLR^xx^ TVR^§§^	TLF^llll^	3.5	Abbott vascular
	PES^†^	332	62.8 ± 10.2	218	92	69.4 ± 13.6	2.76 ± 0.46	14.7 ± 5.7							
SPIRIT IV [50]	EES^*^	2460	63.3 ± 10.5	1664	787	72.3 ± 12.6	—	—	1. Stable, unstable angina; 2. Ischemia with vessel size 2.5–4.25 mm and lesion length ≤ 28 mm	1. Recent MI^‡^,LVEF^x^ < 30 %; 2. LM^§^ bifurcation; 3. lcomplex lesions; 4. Totally occluded vessels; 5. Large bifurcations; 6. Excessive calcification; 7. Tortuosity; 8. Angulation; 9. Thrombus	MAGE^‡‡^ Cardiac death MI^‡^ .ST^††^	TLR^xx^ ID-TLR^#^	TLF^llll^	3	Abbott vascular
	PES^†^	1230	63.3 ± 10.2	833	399	72.0 ± 12.8	—	—							

*Abbreviations*: ^*^:everolimus-eluting stent; ^†^:paclitaxel-eluting stent; ^‡^:myocardial infarction; ^x^LVEF: left ventricular ejection fraction; ^§^:left main; ^ll^:major adverse cardiac events; ^#^:ischemia-driven target lesion revascularization; ^**^:coronary artery bypass graft; ^††^:sent thrombus ; ^‡‡^ :major adverse cardiac events; ^xx^:target lesion revascularization; ^§§^:target vessel revascularization; ^llll^:Target lesion failure

### Results of meta-analysis

#### Three-year outcomes of EES compared to PES [42, 44, 45]

##### Primary outcomes

**MACE**

**Fig. 4:**
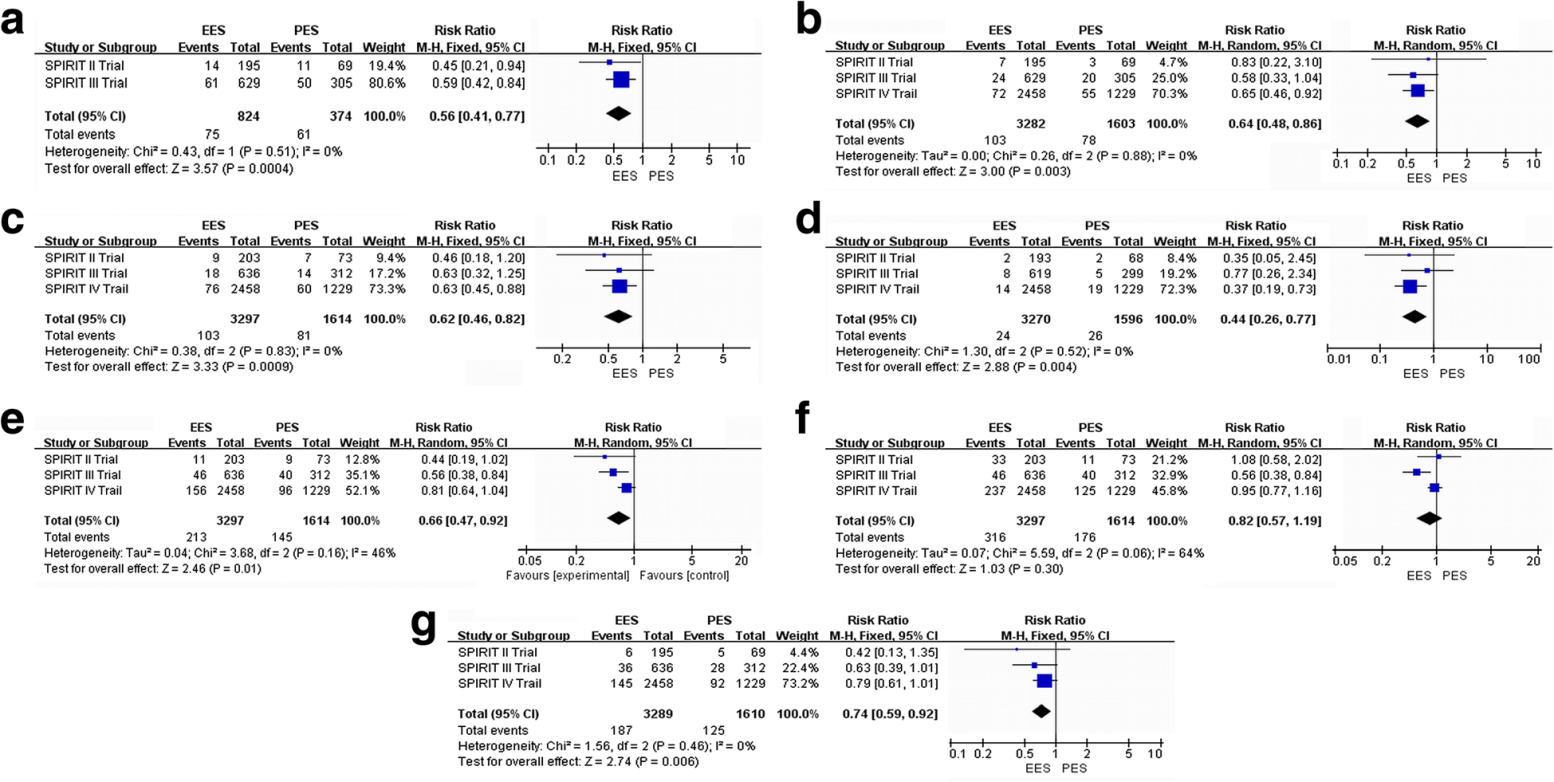
Risk Ratio of 3-year clinical outcomes: the Risk Ratio of (**a**: MACE, **b**: MI, **c**: all cause of death; **d**: ST; **e**: TLR, **f**: TVR, **g**: ID-TLR) at 3-year follow-up associated with EES versus PES

SPIRIT IV Trail [45] did not publish the MACE data of 3-year outcomes of EES compared to PES. Therefore, the incidence of MAGE at all follow-ups was 9.10 % (75 of 824) among patients treated with the EES and 16.31 % (61 of 374) among patients treated with the PES (RR: 0.56, 95 % CI: 0.41–0.77; *P* < 0.05) [42, 44], with no significant study heterogeneity (Chi2 = 0.43; *P* = 0.51; I2 = 0 %) (Fig. 4(a)). There was a statistically significant difference between EES and PES.

##### **MI**

The incidence of MI at all follow-ups was 3.14 % (103 of 3282) among patients treated with the EES and 4.86 % (78 of 1603) among patients treated with the PES (RR: 0.64, 95 % CI: 0.48–0.86; *P* < 0.05) [42, 44, 45], with no significant study heterogeneity (Chi2 = 2.26; *P* = 0.88; I2 = 0 %) (Fig. 4(b)). There was a statistically significant difference between EES and PES.

##### **All-cause death**

The incidence of all-cause death at all follow-ups was 3.12 % (103 of 3297) among patients treated with the EES and 5.02 % (81 of 1614) among patients treated with the PES (RR: 0.63, 95 % CI: 0.46–0.82; *P* < 0.05) [42, 44, 45], with no significant study heterogeneity (Chi2 = 0.38; *P* = 0.83; I2 = 0 %) (Fig. 4(c)). There was a statistically significant difference between EES and PES.

##### **ST**

The Academic Research Consortium’s (ARC) consensus definite/probable ST were totally included for analysis. The incidence of all STs at all follow-ups was 0.73 % (24 of 3270) among patients treated with the EES and 1.63 % (26 of 1596) among patients treated with the PES (RR: 0.44, 95 % CI: 0.26–0.97; *P* < 0.05) [42, 44, 45], with no significant study heterogeneity (Chi2 = 1.30; *P* = 0.52; I2 = 0 %) (Fig. 4(d)). There was a statistically significant difference between EES and PES.

##### Secondary outcomes

**TLR**

The incidence of TLR at all follow-ups was 6.46 % (213 of 3297) among patients treated with the EES and 8.98 % (145 of 1614) among patients treated with the PES (RR: 0.66, 95 % CI: 0.47–0.92; *P* < 0.05) [42, 44, 45], with no significant study heterogeneity (Chi2 = 3.68; *P* = 0.16; I2 = 46 %) (Fig. 4(e)). There was a statistically significant difference between EES and PES.

##### **TVR**

The incidence of TVR at all follow-ups was 9.28 % (306 of 3297) among patients treated with the EES and 10.90 % (176 of 1614) among patients treated with the PES (RR: 0.82, 95 % CI: 0.57–1.19; *P* > 0.05) [42, 44, 45], with a significant study heterogeneity (Chi2 = 5.59; *P* = 0.06; I2 = 64 %) (Fig. 4(f)). There was no statistically significant difference between EES and PES.

##### **ID-TLR**

The incidence of ID-TLR at all follow-ups was 5.68 % (187 of 3289) among patients treated with the EES and 7.76 % (125 of 1610) among patients treated with the PES (RR: 0.74, 95 % CI: 0.59–0.92; *P* < 0.05) [42, 44, 45], with no significant study heterogeneity (Chi2 = 1.56; *P* = 0. 46; I2 = 0 %) (Fig. 4(g)). There was a statistically significant difference between EES and PES.

#### Four-year outcomes of EES compared to PES [47]

In all included studies, only SPRIT II Trail reported 4-year follow-up clinical outcomes. We extracted the data of 4 year-end points and analyzed as following.

##### Primary outcomes

**MACE**

**Fig. 5:**
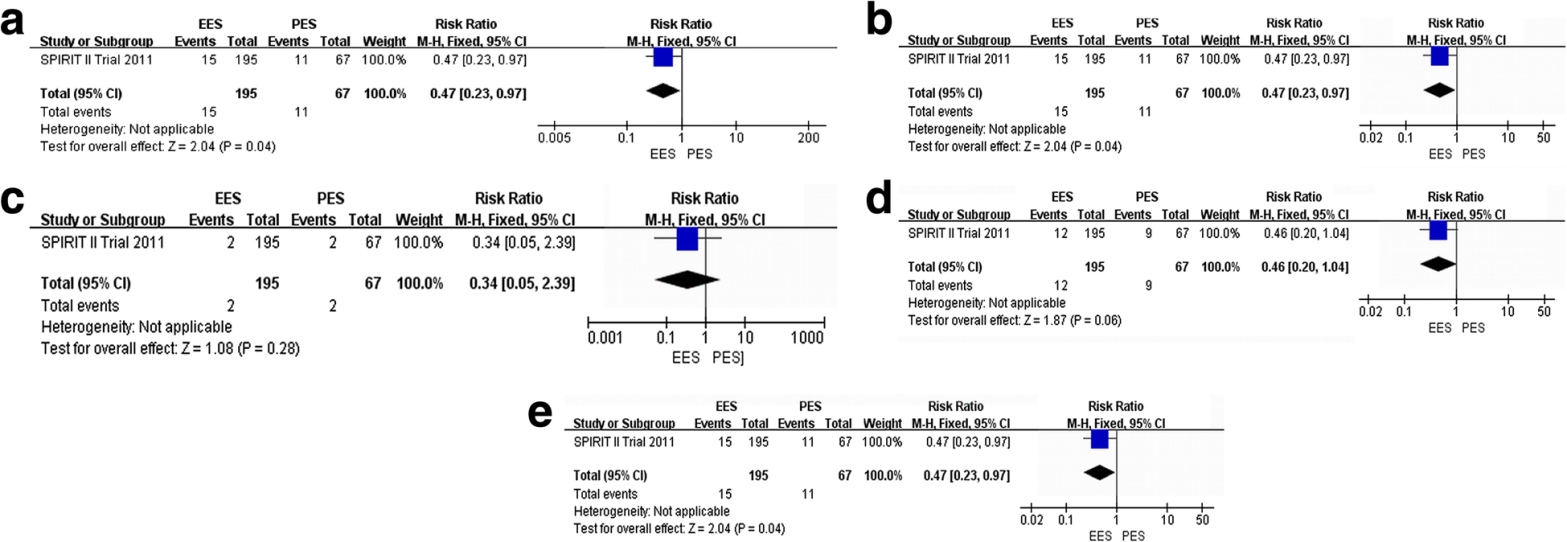
Risk Ratio of 4-year clinical outcomes: the Risk Ratio of **a**: MACE, **b**: MI; **c**: ST; **d**: TLR; **e**: ID-TLR) at 4-year follow-up associated with EES versus PES

The incidence of MAGE at all follow-ups was 7.62 % (15 of 195) among patients treated with the EES and 16.42 % (11 of 67) among patients treated with the PES (RR: 0.47, 95 % CI: 0.23–0.97; *P* < 0.05) [47] (Fig. 5(a)). There was a statistically significant difference between EES and PES.

##### **MI**

The incidence of MI at all follow-ups was 3.59 % (7 of 195) among patients treated with the EES and 7.46 % (5 of 67) among patients treated with the PES (RR: 0.48, 95 % CI: 0.16–1.46; *P* > 0.05) [47] (Fig. 5(b)). There was no statistically significant difference between EES and PES.

##### **ST**

The incidence of ST at all follow-ups was 1.02 % (2 of 195) among patients treated with the EES and 2.98 % (2 of 67) among patients treated with the PES (RR: 0.34, 95 % CI: 0.05–2.39; *P* > 0.05) [47] (Fig. 5(c)). There was no statistically significant difference between EES and PES.

##### Secondary outcomes

**TLR**

The incidence of TLR at all follow-ups was 6.15 % (12 of 195) among patients treated with the EES and 13.43 % (9 of 67) among patients treated with the PES (RR: 0.46, 95 % CI: 0.20–1.04; *P* > 0.05) [47] (Fig. 5(d)). There was no statistically significant difference between EES and PES.

##### **ID-TLR**

The incidence of ID-TLR at all follow-ups was 7.69 % (15 of 195) among patients treated with the EES and 16.42 % (11 of 67) among patients treated with the PES (RR: 0.47, 95 % CI: 0.23–0.97; *P* < 0.05) [47] (Fig. 5(f)). There was a statistically significant difference between EES and PES.

#### Five-year outcomes of EES compared to PES [43, 46]

In all included studies, only SPRIT II, III and IV Trail reported 4-year follow-up clinical outcomes, SPRIT III Trail did not publish relevant data, so we extracted the reported data of 5 year-end points and analyzed as following.

##### Primary outcomes

**ST**

**Fig. 6:**
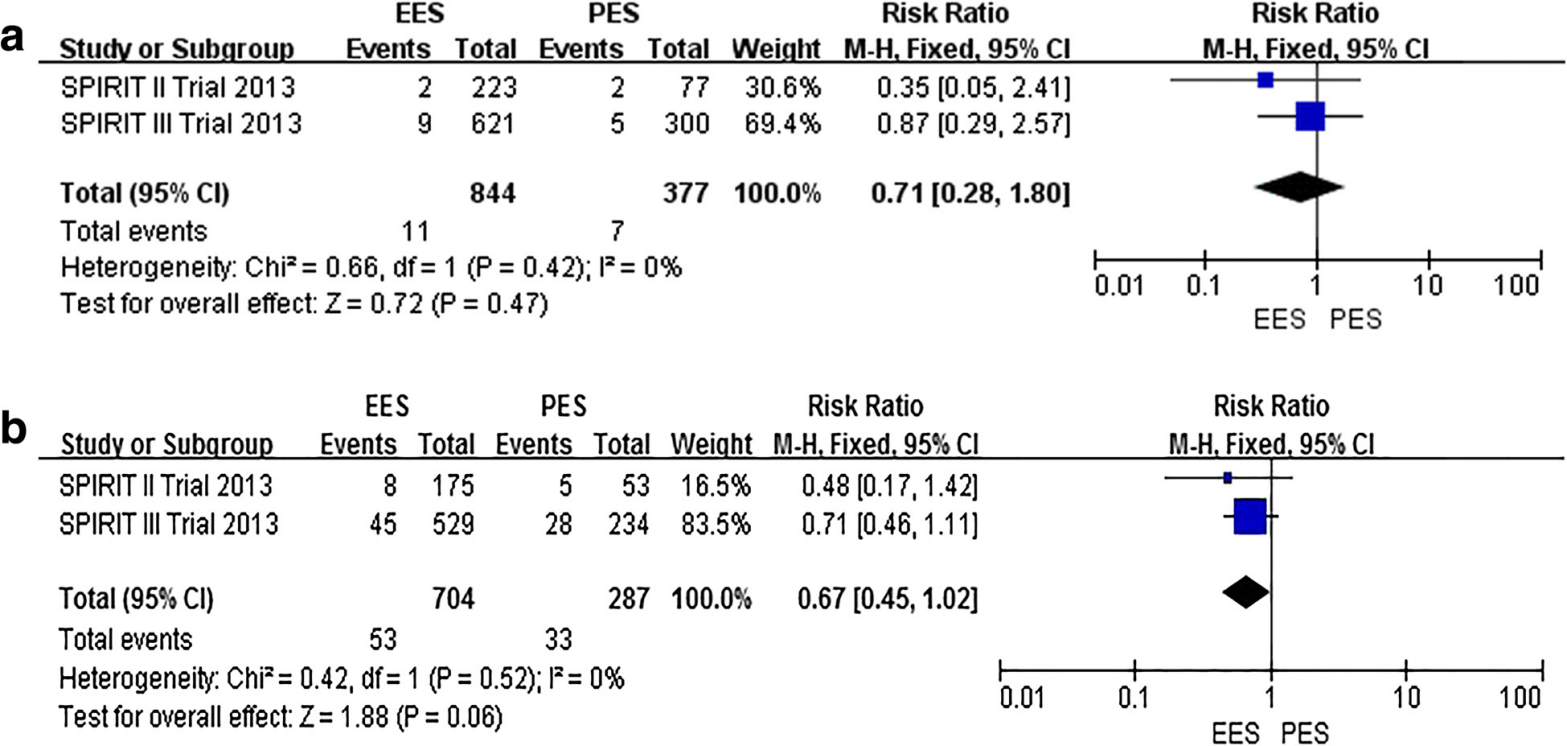
Risk Ratio of 5-year clinical outcomes: the Risk Ratio of **a**: ST, **b**: ID-TLR) at 5-year follow-up associated with EES versus PES

The incidence of definite/probable ARC ST at all follow-ups was 1.30 % (11 of 844) among patients treated with the EES and 1.86 % (7 of 377) among patients treated with the PES (RR: 0.71, 95 % CI: 0.28–1.80; *P* > 0.05) [43, 46], with no significant study heterogeneity (Chi2 = 0.66; *P* = 0.42; I2 = 0 %) (Fig. 6(a)). There was no statistically significant difference between EES and PES.

##### Secondary outcomes

**ID-TLR**

The incidence of ID-TLR at all follow-ups was 7.53 % (53 of 704) among patients treated with the EES and 11.50 % (33 of 287) among patients treated with the PES (RR: 0.67, 95 % CI: 0.45–1.02; *P* > 0.05) [43, 46], with no significant study heterogeneity (Chi2 = 0.42; *P* = 0. 52; I2 = 0 %) (Fig. 6(b)). There was no statistically significant difference between EES and PES.

## Discussions

### Characteristic and quality of included studies

For this meta-analysis, we screened 1320 articles, only 6 studies (three trails) [42–47] which met the inclusion criteria were considered. The randomized trials in our meta-analysis included patients with PCI, and two of which described a method of generating randomness. SPRIT Trails were all single-blinded, but it was not clear whether allocation concealment was used, and reported failing in follow-ups. Risk of bias graph is shown in Fig. 2.

SPIRIT Trails included a proportion of the diabetic population, especially SPIRIT IV trail, which included 32.14 % diabetics [45]. The baseline characteristic of SPRIT Trails were consistent, described safety of eluting stent using the dangers of endpoints such as MACE, MI, TLR,ID-TLR, instead of beneficial indicators, in order to avoid biases in outcome evaluation.

### Clinical significance of this meta-analysis

In recent years, drug-eluting stents have revolutionized interventional cardiology and become an important part of interventional cardiology. New stents through large RCT trials has been verified to be effective, but it should be a long-term follow-up of clinical indicators to evaluate the difference of long-term efficacy and safety of new stents and old stents. Meta-analysis and RCT trials showed that EES could reduce dangerous endpoints, such as MACE, MI, TLR, ID-TLR in ≤ 3 year follow-ups when compared with PES.

The present meta-analysis of RCTs compared EES to PES for PCI with clinical follow-ups from three to 5 years, using multiple endpoints. Based on results of the 3-year clinical follow-up [42, 44, 45] combined dataset, significant reductions with EES compared with PES were observed for the safety endpoints of all-cause death and MACE, MI, and ST, and improved efficacy with reducing rates of TLR, ID-TLR, but similar rates in the two groups of TVR. Only one trail (SPIRIT IV) [47] was included for analyzing 4-year clinical follow-up results, which demonstrated EES could reduce MACE and ID-TLR, which is better than PES, but couldn’t reduce MI, TLR and ST.

The main finding of our meta-analysis is that we demonstrated EES was superior clinical efficacy to the PES in the reduction of ID-TLR and ST by the four [47] and 5-year clinical follow-up [43, 46] combined dataset. However, the differences were not statistically significant. A meta-analysis reported by Ashraf Alazzoni [29] demonstrated that EES was superior to PES in reducing early (0–30 days), late (31–365 days), and very late ST (*>*365 days) with statistically significant difference, but the very late ST group had not to subgroup analysis. It might imply a non-significant trend for reducing ST at a very long term clinical follow up, and that might be related to sample size problem.

### Study limitations

First, the 3-year follow-up clinical trial [42, 44, 45] comparing outcomes demonstrated that a consistent heterogeneity was observed for the TVR endpoints(I2 ≥ 50 %), even when Odds Ratio were used for analysis. A source of heterogeneity might be due to the reported inconsistency in patients’ number of TVR. for example, SPIRIT II Trail [42, 46, 47] clearly illustrated all of TVR numbers included all the numbers of TLR, but SPIRIT III [43, 44] and SPIRIT IV Trail [46] did not clearly state whether the occurrence of TVR number included all TLR patients. However, other key indicators clearly demonstrated that EES had certain superiority to PES by the 3-year clinical follow-up combined dataset.

Second, Spirit Trails were all sponsored by Abbott Vascular, therefore there might be a certain risk of results’ bias.

Third, this meta-analysis of RCT trials were from the same series, due to the relatively limitation of probably same inclusion standard and same researchers, there is a certain risk of results’ bias for long-term efficacy and safety with EES and PES. Therefore, we should include outcomes data of Compare Trail for 3–5 years clinical follow-up, in order to avoid results’ bias mentioned above. Unfortunately, the relevant data of Compared Trail has not been reported so far [37, 38].

Finally, limited databases were searched and only published articles were included, as a result, there is the possibility of no comprehensive trails. As clinical significance of this meta-analysis might be uncertain, larger sample size will be needed to collect, and higher quality of research will be needed.

### Future research directions

It has been reported that elderly, women and types of vascular of Sprite III Trial were analyzed for evaluating a long term clinical endpoints [48, 49]. Considering the bias of present study, it is recommended that future large, randomized clinical trials for diabetic patients should be carried out, and then diabetes will be included for sub-analysis respectively in order to evaluate significance safety of EES and PES in diabetes, which do not continue to further at moment for few original research data. The risk of stent fracture in EES has not been assessed [50], therefore, further studies should focus on that as well.

## Conclusions

The current evidence showed that EES appeared to be safe and clinically effective in patients undergoing PCI comparing to PES in long-term clinical outcomes. However, more randomized data are needed, especially on 4-year follow-ups.

## Role of the funding source

There was no funding source for this study. The corresponding author had full access to all the data in the study and had final responsibility for the decision to submit for publication.

## Abbreviations

EES: everolimus-eluting stent
PES: paclitaxel-eluting stent
PCI: percutaneous coronary interventions
RCT: randomized controlled trial
RR: relative risk
CI: confidence interval
ACS: acute coronary syndrome
BMS: bare metal stents
DES: drug-eluting stents
MACE: major adverse cardiac events
MI: myocardial infarction
TLR: target lesion revascularization
TVR: target vessel revascularization
TLR: target lesion failure
ID-TLR: ischemia-driven target lesion revascularization
ST: stent thrombus
